# Coronary Risk Assessment and Management Options in Chronic Kidney Disease Patients Prior to Kidney Transplantation

**DOI:** 10.2174/157340309788970342

**Published:** 2009-08

**Authors:** Vanji Karthikeyan, Karthik Ananthasubramaniam

**Affiliations:** Division of Nephrology and Transplantation and the Heart and Vascular Institute, Henry Ford Hospital Detroit MI, USA

**Keywords:** Chronic kidney disease, kidney transplantation, renal transplantation, coronary artery disease, risk stratification, stress echocardiography, dobutamine echocardiography, coronary angiography, myocardial perfusion imaging.

## Abstract

Cardiovascular disease remains the most important cause of morbidity and mortality among kidney transplant recipients. Nearly half the deaths in transplanted patients are attributed to cardiac causes and almost 5% of these deaths occur within the first year after transplantation. The ideal strategies to screen for coronary artery disease (CAD) in chronic kidney disease patients who are evaluated for kidney transplantation (KT) remain controversial. The American Society of Transplantation recommends that patients with diabetes, prior history of ischemic heart disease or an abnormal ECG, or age ≥50 years should be considered as high-risk for CAD and referred for a cardiac stress test and only those with a positive stress test, for coronary angiography. Despite these recommendations, vast variations exist in the way these patients are screened for CAD at different transplant centers. The sensitivity and specificity of noninvasive cardiac tests in CKD patients is much lower than that in the general population. This has prompted the use of direct diagnostic cardiac catheterization in high-risk patients in several transplant centers despite the risks associated with this invasive procedure. No large randomized controlled trials exist to date that address these issues. In this article, we review the existing literature with regards to the available data on cardiovascular risk screening and management options in CKD patients presenting for kidney transplantation and outline a strategy for approach to these patients.

## INTRODUCTION

Cardiovascular (CV) disease is the leading cause of death in patients with chronic kidney disease (CKD). This includes patients on dialysis, those wait listed for transplant and those with kidney transplantation (KT), and accounts for 42% of all deaths in this group [1]. In a study of 23, 546 adult first KT recipients in the United States Renal Data System between Jan 1995 and Sep 1997, 4.6 % died in the first post transplant year, and cardiac causes were the leading cause of death, accounting for 27 % of all deaths [2]. Over half of these patients die with a functioning allograft, resulting in inefficient use of this scarce resource. Therefore, aggressive CV risk screening and management before KT has become a priority. 

Though the overall survival is increased by KT, there is an initial increase in mortality, soon after KT, and the actual survival benefit of KT occurs beyond 250 days of transplantation [3]. Hence, it is obvious that patients should survive beyond this period in order to be benefited from this whole process. CV risk screening for KT is unique in that it needs to assess not only the perioperative risk, but also should ideally assess the CV risk beyond that period and into the early years of transplantation for the above mentioned reason. This review will primarily focus on assessing coronary artery disease (CAD) risk in pre-KT CKD patients.

## EPIDEMIOLOGY AND PATHOPHYSIOLOGY OF CAD IN PRE-KT PATIENTS

CAD is a frequent co morbidity in patients with CKD, with a prevalence varying from 24% in young patients without diabetes to 85% in elderly hemodialysis patients with diabetes [4, 5]. Diabetes is a leading CV risk factor and is considered to be a CAD risk equivalent and 40 -50% of patients on dialysis are indeed diabetics. Many conventional cardiac risk factors such as dyslipidemia, smoking and hypertension are less predictive of CAD in renal failure [6]. The Framingham risk score underestimates the CV events predicted in KT patients [7].

CKD population has additional CV risk related to non traditional factors including microalbuminuria, uremia, hyperuricemia, calcium – phosphorus disorder associated calcification, inflammation and hyperhomocysteinemia. Pre-KT CV risk factors often persist after KT and can worsen in the post transplantation period resulting in accelerated atherosclerosis. Anti rejection therapy used in the post transplant period such as steroids, calcineurin inhibitors and sirolimus increase the development of or worsening of preexisting hypertension, dyslipidemia, hyperuricemia, weight gain and glucose intolerance. All these factors make KT candidates a unique population that may require a different CV risk screening strategy as well as management.

## CLINICAL PREDICTORS OF CAD IN KT CANDIDATES

Presence or absence of chest pain does not correlate well with CAD in CKD patients [8]. Severe CAD is common in asymptomatic patients with CKD, likely because of autonomic dysfunction secondary to uremia and diabetes [8]. Braun *et al. *[9] reported that up to 75% of the patients with CAD undergoing hemodialysis did not have typical angina, making silent CAD three times more prevalent than in the Framingham study population [10]. A large proportion of these patients lead a sedentary lifestyle as a result of reduced exercise capacity which also contributes to the lack of symptoms. The reduced exercise capacity is a result of muscle fatigue, anemia and a generalized feeling of being ill after dialysis likely secondary to rapid fluid and electrolyte shifts. As a result, a good proportion of patients live with functional status below 4 METS, making assessment on clinical grounds difficult. The converse, which is angina without CAD on angiography has also been reported to occur in up to 50% of all patients [11, 12]. Reasons for symptoms in the absence of epicardial CAD include anemia, reduced vasodilator reserve, microvascular disease and supply demand mismatch resulting from severe left ventricular hypertrophy (LVH) that is seen in about 70 % of patients with CKD [13]**. **LVH can lead to increased oxygen demand and subsequently to angina, even in the presence of normal coronary arteries. Rostand *et al. *[11] showed that 27% of dialysis patients who presented with angina did not have evidence of CAD on angiography [14]. The sensitivity and specificity of angina for predicting angiographic CAD was 65% and 66% respectively, and all patients with angina but no major epicardial CAD had LVH [14]. For all these reasons, chest pain has poor sensitivity and specificity for predicting CAD in CKD patients.

Gowdak *et al. *[15] sought to determine clinical predictors of significant CAD (coronary stenosis ≥ 70%) in high-risk KT candidates. Clinical evaluation and coronary angiography (CA) were performed in 301 hemodialysis patients with diabetes (type 1 or 2), evidence of CV disease, or age ≥50 years and they were followed-up for 22 months (median). Of all the risk factors studied, prior MI, peripheral vascular disease (PVD) and diabetes were significantly associated with angiographic CAD and future clinical events. They noted a direct relationship between these clinical variables and the prevalence of significant CAD. When all 3 risk factors were present, the prevalence of CAD (> 70 % stenosis) was 100%, compared to 25.7%, in those in whom all three factors were absent. The study selected out and identified patients in whom the benefit of CA may outweigh the risks of the procedure. This model also favors non invasive testing in patients with no major risk factors which helps to avoid indiscriminate use of CA. These clinical variables not only predicted CA stenosis > 70%, but also fatal and nonfatal CV events. Compared to those with no clinical predictors, the composite incidence of fatal and nonfatal CV events was twofold higher in patients with diabetes, fourfold in patients with PVD, and sixfold in patients with previous MI. Similarly, incidence of CV mortality was increased threefold in patients with diabetes, sixfold in those with PVD, and threefold in patients with a history of MI. They also found that risk analysis has similar sensitivity (80%) and negative predictive value (NPV) as CA in predicting cardiac events.

## CARDIAC SCREENING FOR KT: AN OVERVIEW

Conflicting data exists on the best method of cardiac screening and risk stratifying KT candidates. Some studies have suggested that CA may be the best screening method [16]. Other studies have shown that simple clinical risk stratification as well as non invasive testing such as with stress single photon emission computed tomography (SPECT) can accurately predict perioperative and post transplant cardiac events [17]. Furthermore a meta analysis of myocardial perfusion imaging studies in CKD patients [18] showed that an ischemic study predicted a sixfold increase in MI and fourfold risk of death. Fixed defects were also predictive of increased cardiac death. As with other stress tests a negative perfusion imaging study carries more favorable prognostic value in predicting lower perioperative cardiac events.

Dobutamine stress echocardiography (DSE) has also been studied in this population, but found to have a wide range of sensitivities from 38 to 95% and specificity from 71 to 96% [19, 20]. The differences in the results in these studies are likely related to the differences in the study population, small sample sizes and lack of prospective randomized design, and differences in the medical therapy of CAD instituted successfully.

The American College of Cardiology/American Heart Association (ACC/AHA) guidelines for perioperative evaluation and management has been revised and published in 2007 [19]. The Revised Cardiac Risk Index published by Lee *et al. *[21] is considered the preferred clinical risk assessment tool to identify patients who may require additional evaluation. Renal insufficiency is part of this risk index and has been highlighted as a cardiac risk factor in the recent guidelines. Interestingly no specific recommendations regarding risk stratification strategies for pre-KT assessment are made reflecting lack of well designed studies.

Guidelines for cardiac screening in KT candidates are largely based on data from the non uremic population. The ACC/AHA perioperative assessment guidelines [19] suggest that patients with ≥ 3 clinical risk factors and/or poor functional capacity (< 4 METS equivalent activity level) being evaluated for vascular surgery can be screened using noninvasive cardiac testing (class 11a). Those with 1-2 clinical risk factors and/or poor functional capacity going for intermediate-risk non-cardiac surgery may be considered for noninvasive testing (Class11b). The American Society of Transplantation recommends [22] that patients at high risk, e.g. renal disease from diabetes, prior history of CAD, or ≥2 risk factors, should have a cardiac stress test. Risk factors included here are: a prior history of CAD, men ≥45 or women ≥55 years, CAD in a first degree relative, current cigarette smoking, diabetes, hypertension, fasting total cholesterol > 200 mg/dl, high density lipoprotein cholesterol < 35 mg/dl and LVH. These guidelines recommend that those with positive cardiac stress test should undergo CA [23]. The sensitivity and specificity of non-invasive tests in pre-KT CKD patients is lower than that in the general population [16, 24, 25]. Some transplant centers have adopted a policy of direct diagnostic CA in high risk patients, despite the absence of convincing evidence to support routine invasive stratification. 

In addition to the unanswered questions regarding the initial screening strategy for CAD in KT candidates, there are also uncertainties regarding periodic screening of wait listed patients. A patient may have minimal CAD at the time of initial evaluation, but then develop significant disease while waiting on the KT list. Since CKD is associated with activation of systemic inflammation and increased oxidative stress, this sets the stage for accelerated atherosclerosis seen in this patient group [26]. It is not clear how frequently to retest them, whether re-testing is of value or is periodic clinical evaluation sufficient. No randomized controlled trials exist to date addressing all these issues.

## NON INVASIVE CARDIAC EVALUATION IN PRE-KT CKD PATIENTS: A COMPARATIVE OUTLOOK OF DIFFERENT METHODOLOGIES

### a. Electrocardiogram (EKG)/Exercise Electrocardiography (Treadmill)

An abnormal EKG is very common in CKD patients and was found in 46% of patients in one study [8]. Voltage criteria for LVH, T wave changes and bundle branch blocks account for most of the baseline abnormalities. This high prevalence of abnormal baseline EKG in renal failure has been described by several authors [27, 28] and is thought to be due to LVH, volume overload and electrolyte abnormalities typically seen in CKD patients. Baseline EKG abnormalities are much rarer in the general population, occurring in only 8.5% of men and 7.7% of women [29]. Sharma *et al. *[8] found abnormal baseline EKG to be an independent predictor of angiographically proven CAD in 125 KT candidates all of whom underwent CA. Its sensitivity was 77% but specificity only 58%. Exercise EKG was not predictive of CAD in this group of patients [8]. Others authors found that neither resting nor exercise EKG was predictive of CAD [14]. Because of the high prevalence of non specific abnormalities in baseline EKG and the conflicting literature on its usefulness, neither resting nor exercise EKG is accepted as a useful screening tool in KT candidates undergoing cardiac evaluation.

### b. Cardiac Single Photon Emission Computed Tomography (SPECT)

The sensitivity and specificity of non invasive tests to detect CAD in CKD patients are highly variable and are often below 70% [14, 30]. Marwick *et al. *used a control group in their study showed that the sensitivity of dipyridamole thallium scintigraphy for detecting CAD was 95 % in control group but only 37% in dialysis patients with a modest specificity of 73% using a 50% stenosis detection on CA [25]. Using a quantitative CA lesion severity definition of 70% cross sectional narrowing, Boudreau *et al. *[31] reported 86% sensitivity and 72% specificity for intravenous dipyridamole planar thallium. Vandenberg *et al. *[24] retrospectively compared pharmacologic stress thallium scintigraphy with CA and demonstrated 53% sensitivity and 73% specificity compared with a visually estimated stenosis of 50% or greater and 62% sensitivity and 76% specificity for stenosis of 75% or greater. The lower sensitivity in CKD population has been attributed to several factors. CKD patients have higher levels of basal adenosine resulting in a high resting coronary flow. Pharmacologic myocardial perfusion imaging uses dipyridamole (which works by increasing endogenous adenosine levels) or adenosine as vasodilator to induce stress by challenging the flow reserve. The higher resting blood flow in CKD patients blunts the flow reserve challenge and thus can compromise the sensitivity of SPECT imaging by decreasing heterogeneity of radioisotope uptake which forms the basis of detection of CAD by this modality [25]. Also many pre-KT patients are on multiple anti-hypertensive’s, many of which are also anti-anginals (beta-blockers, calcium blockers, nitrates). These agents are known to decrease sensitivity of stress SPECT imaging [32, 33] by reducing ischemic burden. Despite these limitations, SPECT imaging has been recently shown to provide useful information for pre-KT risk assessment. Patel *et al. *[34] studied 600 patients undergoing pre-KT evaluation and among these 174 had SPECT imaging done. In these patients adverse outcomes were predicted by an abnormal SPECT (the only multivariate predictor) whereas event free survival was 97% in patients with a normal SPECT over a 42 month follow-up period. Thus, a negative SPECT carries a very favorable prognostic value in the short and long term prediction of low cardiac events used as an endpoint. A positive SPECT also carries adverse outcomes and warrants aggressive medical therapy and consideration of revascularization if ischemic burden is high.

### c. Dobutamine Stress Echocardiography (DSE)

DSE is a well established modality for detection and prognostication of CAD [35]. Prior studies have shown consistently the excellent negative predictive value of a normal DSE in patients undergoing major surgery [36, 37]. 

De Lima *et al. *[16] prospectively studied KT candidates subjecting them to CA, clinical risk stratification and 2 non invasive modalities of testing - DSE and dipyridamole SPECT with tech 99 MIBI, and followed them for a mean of 26 months for cardiac events. Patients were chosen based on the presence of at least one risk factor: age ≥ 50 years, DM, angina, prior MI or stroke, LV dysfunction or extra cardiac atherosclerosis. One hundred and twenty six patients underwent one or more of the 3 tests and 88 patients underwent all 3 tests. The results of the non invasive tests and clinical risk stratification correlated significantly with the degree of CAD, defined in this study as ≥ 70 % coronary stenosis by visual estimation. However, 30% of patients with negative results on both non invasive studies had critical coronary stenosis on CA. The sensitivities of DSE, SPECT and clinical risk stratification were low, between 35 and 64%, NPV < 70%. DSE and SPECT results did not predict future cardiac events. Simple clinical risk stratification was able to better predict future cardiac events than the two non invasive tests in this study. 

Reis *et al. *[38] also studied DSE in KT candidates and used only fatal events as end points during a follow-up period of 12 ± 6 months. Twenty-nine of 97 patients studied had inducible ischemia by DSE (30%). DSE had a positive predictive value of 14% and a negative predictive value of 97% for 1-year survival indicating the more robust predictive value for a normal DSE. On the other hand, several studies have found DSE to be able predict CAD in KT candidates [20]. A positive DSE has been identified as an independent predictor of severe CAD [8, 20]. Herzog *et al. *[20] compared DSE and CA in 50 patients evaluated for KT. The sensitivity and specificity of DSE was tested for detection of quantitative coronary angiography (QCA) stenosis of 50%, 70 % and qualitative visual estimation for stenosis > 75%. Twenty of 50 DSE tests were positive for inducible ischemia. The sensitivity and specificity of DSE for CAD diagnosis were respectively 52% and 74% for QCA stenosis of ≥ 50%, 75% and 71% for QCA stenosis greater than 70%, and 75% and 76% for stenosis greater than 75% by visual estimate. On long-term follow-up (22.5 ± 10.1 months), 6 of 30 patients (20%) with negative DSE results and 11 of 20 patients (55%) with positive DSE results had a cardiac death, MI, or coronary revascularization. During a mean follow of 22 months, for the combined end points of cardiac death, MI, or coronary revascularization, a positive DSE result was associated with significantly worse event-free survival. The event rate in positive and negative DSE results was 55 % and 20% respectively. In this study, a positive DSE was associated with angiographic CAD and future cardiac events. More importantly, a normal DSE predicted event free survival even in those with angiographic CAD. Others such as Bates *et al. *[39] have also reported prognostication with DSE in that 9 of 20 patients (45%) with a positive DSE and 2 of 33 patients (6%) with a negative DSE had a cardiac event in a follow-up period of 813 **±** 395 days, a statistically significant difference.

More recently the role of DSE in cardiac risk stratification was evaluated by the us (authors) at our institution in a retrospective study of 149 patients who underwent DSE prior to KT [40]. This study conducted on a predominant African American population again showed that DSE had 37.5% sensitivity, 95.3% specificity, 33.3% positive predictive value, and 96.1% negative predictive value for major cardiac events in the first year after KT. First-year post transplant event rates were 4.0% versus 30% (*P *< 0.001) for patients with negative and positive stress echo, respectively. This remained true regardless of the need for revascularization procedures, as shown in our study where among the 12 patients who were subjected to CA (all 10 patients with positive DSE and 2 others with very sub maximal stress levels), only one patient needed revascularization. This reemphasizes the importance of non-occlusive atheromata as being important targets for aggressive medical therapy and plaque stabilization. A positive DSE thus identifies a high risk group of individuals who regardless of CA results have increased cardiac events. 

In general DSE is more specific than SPECT for CAD diagnosis as it uses ischemia as the endpoint of CAD compared to flow heterogeneity in SPECT which renders the latter more sensitive but less specific. But DSE has its limitations in that presence of underlying LVH with resultant small intra cavitary volume occurring at peak dobutamine stress that may obscure the detection of minor wall motion abnormalities [41]. In particular, concentric remodeling of the left ventricle was found to be the most frequent cause of false negative results with DSE in one study [42]. It is felt that blunted rise in end-systolic wall stress may be one of the mechanisms for this underestimation of CAD. Another reason for a false negative DSE is lack of achievement of target heart rate responses. Although the prognostic implications regarding sub-maximal DSE has been conflicting with some studies suggesting adverse outcomes [43, 44] and others favorable outcomes, [45], below target heart rates can decrease detection of anatomical CAD. In this regard, CKD patients have most of the characteristics which may decrease the sensitivity of DSE for CAD detection. They have a high incidence of HTN, LVH and also a high incidence of sub maximal heart rates during DSE [40]. Thus, all of these factors need to be kept in mind when utilizing and interpreting DSE in CKD patients.

### d. Coronary Angiography (CA)

CA being an invasive procedure, the benefits have to be weighed against the risk in any individual. Some authors advocate CA in all high risk CKD patients, particularly diabetics being evaluated for KT. In a study by Sharma *et al., *of 125 renal transplant candidates who underwent CA [8], 44% of diabetics had severe CAD, defined as luminal stenosis >70% by visual estimation in at least one epicardial artery and mortality was significantly higher in this group. However, only 20% of non-diabetics had severe CAD. Overall, 36/125 patients had >70% stenosis, but only 15/125 (12%) underwent revascularization and CA did not lead to active intervention in 88% of patients. To date, there is only one randomized prospective study assessing revascularization* vs. *medical therapy in KT candidates done in early 1990’s. Manske *et al. *[46] subjected 151 consecutive asymptomatic insulin-dependent diabetic KT candidates to CA. Thirty one patients had at least one coronary artery stenosis greater than 75%. Patients with angina or a left ventricular ejection fraction below 35% and significant three-vessel disease were excluded. Patients with coronary artery lesions judged to be hemodynamically significant and suitable for revascularization, were then randomized to 2 strategies. Twenty six were randomized to either medical therapy (13 patients), consisting of calcium channel blockers (CCB) and aspirin* vs. *revascularization (13 patients). Ten of 13 medically managed and 2 of 13 revascularized patients had a cardiovascular endpoint within a median of 8.4 months of coronary angiography. They concluded that revascularization of angiographically significant coronary artery stenoses in asymptomatic diabetic KT candidates may decrease the incidence of cardiac events. This study does not address the issue of which test will be ideal to detect CAD, as no noninvasive testing was done. Although it supports revascularization in these patients with significant CAD, it does not support doing routine CA in all KT candidates. Only 31 out of 151 (20.5%) had stenosis > 75% in this study by CA. Nearly 80% had insignificant CAD and therefore unnecessary CA. Uniform medical treatment between the two groups could not be confirmed. Also, CCB were used as anti ischemic therapy, not betablockers for fear of aggravating hypo-glycemic unawareness. Statins were not used in all patients and current aggressive medical therapy for stable CAD as shown in the COURAGE trial [46] was not done at that time. It is possible that the medical therapy group would have done better with betablockers and high dose statins. Finally this trial focused on diabetics and is not applicable to non-diabetic CKD patients. Subsequent to this trial there are no published randomized data in over a decade comparing pre-KT CKD patients on aggressive medical regimen (aspirin, betablockers, statins) to revascularization strategies.

More recent studies have addressed whether pre surgical CA and revascularization are warranted prior to vascular and other major surgeries. The Coronary Artery Revascularization Project (CARP) [47] randomized patients with greater than 70% coronary stenosis who were scheduled to undergo elective vascular surgery, to coronary revascularization* vs. *no revascularization. No difference in primary outcome (mortality) or secondary outcome measures (MI, stroke, renal failure, and limb loss) were seen during the follow up period. This is the first and largest randomized trial to date showing that routine revascularization prior to major vascular surgery was not associated with improved outcomes. The DECREASE-V pilot randomized study [48] looked at noninvasive versus prophylactic revascularizations strategies in patients with 3 vessel CAD undergoing major vascular surgery. It showed no advantage of prophylactic revascularization in patients with stable CAD with composite endpoint of all-cause mortality and MI at 30 days and 1 year. It is important to note that both these trials were underpowered for outcomes. Nevertheless the 2007 ACC /AHA guidelines have also recommended that prophylactic revascularization is not warranted prior to non-cardiac surgery in stable CAD patients. The ACC/AHA perioperative guidelines and the results of these 3 trials (CARP, COURAGE and DECREASE –V)(11,43-45) reemphasize that aggressive medical therapy for stable CAD is a very viable option in the perioperative setting. 

Can the ACC/AHA peri-operative assessment guidelines and the results of the above trials be applied to pre-KT assessment? The answer is “not clear” and the authors believe that evidence from existing guidelines cannot be readily extrapolated to KT candidates for the following reasons: In the general population the preoperative and postoperative risk are not significantly altered as a sequelae of surgery. However following KT, patients are exposed to a postoperative milieu that promotes development or worsening of accelerated atherosclerosis both due to presence of persistent abnormal renal function [49] and the adverse effect of immunosuppressive drugs [49]. Thus, uncorrected and unrecognized CAD could get worse after KT leading to complications including fatal CV events, wasting a valuable and limited resource, the transplanted kidney [2]. Furthermore, subjecting patients to CA soon after KT carries the risk of exposure of transplanted kidney to radiocontrast dye with risk of worsening renal function. Thus addressing significant CAD prior to KT may carry a dual advantage of avoiding cardiac morbidity and mortality and preventing CA after KT. Most centers involved in KT follow the AST guidelines which is to perform a noninvasive screening test in those with cardiac risk factors and CA is reserved for patients with abnormal stress test results. Although some centers perform routine CA, there is no convincing evidence that routine CA is justified in all KT candidates, as only a minority of these diagnostic catheterizations lead to any revascularization in the pre-KT CKD patient [8, 40]. Furthermore contrast nephropathy, defined as a serum creatinine increase of greater than 25% when measured 48 hours after radiocontrast exposure, can occur in up to 50% of azotemic diabetic patients undergoing CA [50]. Careful thought and discussion with the pre-KT patient is needed prior to CA particularly when using it as a screening test. The good news is that the risk of loss of any residual renal function in patients on peritoneal dialysis, undergoing coronary angiography has been studied prospectively [51] and retrospectively [52] and appears to be quite small. No accelerated decline in renal function was noted in stable peritoneal dialysis patients. So adopting an initial non-invasive strategy followed by CA as deemed necessary may avoid unnecessary exposure to invasive procedures and contrast.

### e. Coronary Computed Tomography Angiography (CCTA)

It is well known that CKD patients, particularly those on hemodialysis have a higher burden of coronary calcification than the general population [53]. This along with the concern of exposing CKD patients to radiocontrast dye has essentially excluded these patients from CCTA studies. To our knowledge there is no published study employing CCTA to evaluate CAD in CKD patients on dialysis. All data using CCTA in advanced CKD patients are limited to calcium burden assessment which is a non-contrast procedure. Preliminary data has just been published in abstract form by our group testing the Safety, Feasibility and Interpretability of CCTA in pre-KT patients on hemodialysis or peritoneal dialysis [54]. This study looked at 28 patients on dialysis undergoing cardiac risk assessment before KT. Interestingly 35% of patients had a zero calcium score and interpretability was excellent for conclusively excluding significant proximal-mid coronary disease in all 3 vessels and in the left main territory. Furthermore CCTA was able to conclusively exclude significant CAD in 7/11 patients with a submaximal dobutamine echo thus avoiding another stress test or CA. CCTA was well tolerated by all patients and there were no study related complications. Further studies are needed to evaluate if a certain subgroup of CKD patients on dialysis can be offered CCTA as a noninvasive screening tool for CAD.

## MANAGEMENT STRATEGIES IN THE PRE-KT CKD PATIENT

### Medical Therapy

There is sparse literature focusing on specific medical therapy to optimize and reduce perioperative risk in CKD patients going for KT. Most of the cardiac protective therapies such as aspirin, betablockers and statins used in the perioperative period are based on evidence from the general population studies. Since CKD is considered a coronary risk equivalent and is associated with multiple cardiac risk factors, use of these agents may seem a rational approach but has so far not been well studied. We will focus on three of the most common medication strategies employed for reducing cardiac risk, namely aspirin, betablockers and statins.

### Aspirin

The role of aspirin in preventing atherothrombotic events in high risk patients is known [55, 56]. However its role in primary cardiac event prevention strategy in CKD patients has not been conclusively demonstrated. Haemostatic abnormalities and uremic platelet dysfunction increase as kidney function declines. Thus possible benefits of anti-platelet therapy may be offset by a higher absolute risk of bleeding [57, 58]. Of the 2632 hemodialysis patients in the anti-platelet trial meta analysis [55] 46 bleeds occurred but not of sufficient number to demonstrate a risk. In favor of aspirin a 41% reduction in MI, stroke and vascular deaths were noted. The United Kingdom Heart and Renal Protection placebo controlled study [59] tested the safety and efficacy of simvastatin 20 mg daily and 100 mg /day of modified release aspirin in 448 predialysis, dialysis and KT patients. There was a threefold increase in minor bleeds but no statistically significant increase in major bleeds. In summary, although the risk of atherosclerotic disease in CKD patients would suggest aspirin as being a beneficial strategy for primary prevention, randomized trials are lacking for this and the concern for bleeding exists. Thus this risk should be discussed with the patient prior to initiation of aspirin for primary prevention. In pre-KT patients with established CAD, prior MI, stroke or PVD low dose aspirin (81mg/day) should be used as benefits outweigh risks. The evidence for use of clopidogrel is lacking for both primary and secondary prevention of atherosclerotic disease in CKD and hence should be reserved for standard indications where combination antiplatelet therapy may be needed (i.e.: post stent implantation) or when patients are aspirin intolerant.

### Betablockers

The main premise for prescribing betablockers in the peri-operative period is to establish a favorable balance of myocardial oxygen supply-demand. Although prior studies have demonstrated beneficial effects [60-62] more recent studies have suggested no benefit [63, 64] with one indicating increasing mortality (POISE trial) [63]. The use of cardio protective medications is also uncommon in pre-KT CKD patients and only 20 to 45% of high-risk patients received either angiotensin-converting enzyme inhibitors (ACEI), betablockers, or lipid-lowering medications at the time of wait-listing [8]. More disappointing is that betablockers and ACEI are not used significantly more often even in those CKD patients with CAD [8].

It is also still unclear which beta blocker should be used, when they should be initiated and how long they should be continued. There may be two facets to consider when using betablockers in KT candidates: 1. an acute effect on myocardial oxygen demand, heart rate and blood pressure brought about by initiation hours prior to surgery and 2. longer term plaque stabilizing effect when started days prior to surgery. In particular the DECREASE –I trial started bisoprolol (longer half-life) an average of 37 days prior to surgery with careful up titration and was associated with a tenfold decrease in perioperative events [61]. This is in contrast to the POISE trial which gave high dose controlled release metoprolol just prior to surgery with maximum doses of 400 mg achieved 1 day after surgery. POISE showed that although cardiovascular events were decreased by beta-blockers, overall mortality was increased likely due to a higher incidence of ischemic stroke. Thus, although no particular beta blocker/regimen can be specifically recommended at this time, we suggest that KT candidates be treated by the strategy based on the ACC/AHA guidelines (11): 1. Betablockers should probably be initiated if possible in all patients as they have a higher risk for atherosclerosis, more importantly if they have a positive stress test. 2. Patients on betablockers should continue on it. 3. Tight heart rate control (HR 60-65 beats per minute) in the perioperative period be done to decrease cardiac events. 4. Betablockers should be started at least 1-3 weeks prior to surgery and up titrated to get adequate heart rate control (HR< 65). We recommend that they be continued in the perioperative period given beneficial effects on long term outcomes [60] and because abrupt discontinuation can be associated with increased adverse outcomes [65].

### Statins

Although CKD patients on hemodialysis have a high CV morbidity and mortality the evidence for lipid lowering agents in modulating this risk is at best equivocal. Initial observational data favoring reduction in CV and total mortality with statin therapy has been published [66, 67] but subsequent studies have not been positive. There are several reasons as to why lowering LDL cholesterol may not be as beneficial in advanced CKD patients. In dialysis patients a negative association has been demonstrated between cholesterol and mortality [68, 69]. Only approximately one quarter of the cardiac mortality in CKD is attributable to acute MI [70]. This was shown in the 4D study which evaluated 1255 Type 2 diabetics on hemodialysis and randomized them to 20 mg atorvastatin or placebo [71]. In contrast to the observational data [66, 67] this study showed no significant difference in composite endpoint of CV death, nonfatal MI or stroke. Concerning was that atorvastatin seemed to increase the risk for fatal stroke. A significant proportion of the deaths were actually attributed to sudden cardiac death in both groups (59% of deaths) suggesting that death may be driven by dysrhythmia not modifiable by statins. Other non-statin agents have also been studied. The OPACH trial was a randomized double-blind placebo controlled trial testing 206 hemodialysis patients with either 1.7 grams/day of omega-3 fatty acids or an olive oil placebo. Although primary endpoint of CV events or death was non significant, a 70% reduction in MI was noted [72]. 

The role of statins in advanced CKD patients may be better clarified by 2 ongoing trials, one in 2700 hemodialysis patients, testing 10mg of rosuvastatin versus placebo (the AURORA study) [73], and the other, Study of Renal and Heart Protection (SHARP) one arm of which is comparing 20 mg of simvastatin to 20mg of simvastatin plus eztemibe in 3000 hemodialysis patients [59]. Until then, given the CV risk profile of CKD patients we believe the following approaches could be considered. 1. For patients with documented CAD, statins should be initiated to attain target LDL levels of < 70 mg/dl. 2. In CKD patients with no known CAD, statins should be strongly considered if LDL > 100, and targeted to less than 100 mg /dl. 3. For CKD patients with LDL levels < 100 mg/dl, if CAD or other atherosclerotic vascular disease is present or if ischemia is detected on stress testing, low dose statins can be initiated given pleiotropic effects of statins on plaque stabilization and atherosclerotic vascular disease mitigation independent of LDL lowering [74, 75]. Since this manuscript primarily focuses on CKD patients, statins with limited renal excretions such as atorvastatin and fluvasatin may be good initial choices. Combination therapy with fibrates needs to avoided as they are metabolized through the kidney and are not recommended in patients with GFR < 15 ml/min/1.73 sq m. by the National Lipid Association. If triglycerides are high, omega-3 fatty acids 3-4 grams /day can be used.

### Coronary Revascularization

When revascularization is contemplated in a KT candidate with significant CAD, several important issues need to be addressed. If percutaneous coronary revascularization is planned, it may warrant use of drug eluting stents to reduce re-stenosis in this high risk population. The use of drug eluting stents poses a problem if a patient is to be listed for KT as clopidogrel and aspirin therapy are currently recommended to be continued uninterrupted for at least one year and maybe longer after the intervention [19]. Premature interruption of these agents for elective KT poses unnecessary risk of acute stent thrombosis and MI which is deleterious to the transplanted kidney and disastrous for the patient as mortality rates are high. Thus when PCI is contemplated, consideration for bare metal stents (requiring only 30-45 days of uninterrupted dual anti-platelet therapy with subsequent option of clopidogrel discontinuation), or plain old balloon angioplasty should be considered. If anatomy is suitable for bypass surgery this option should be discussed with the patient. In our institution as part of pre CA discussion the CKD patient and nephrologist are made aware that if a drug eluting stent is placed the patient will be off the KT waiting list at least for 1 year and then reevaluated by cardiology prior to relisting. Aspirin is recommended to be continued uninterrupted even during KT if clopidogrel is stopped. 

**Fig. (1):**
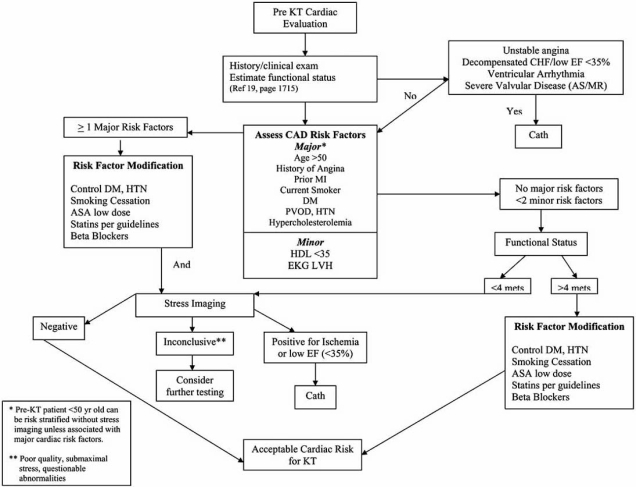
Proposed algorithm outlining cardiac risk stratification approach in pre –KT patients. (KT= kidney transplant).

In summary after review of all available evidence and based on our institutional experience we have outlined an algorithm of how to approach a KT candidate with regards to coronary risk assessment (Fig. **1**).

## CONCLUSION

Well designed prospective randomized trials are urgently needed to identify the best cardiac screening modality in KT candidates with low, intermediate and high clinical risk profiles. Specific guidelines are needed to regulate the management of CV risk factors both before and after KT. Ideally this should be based on strong medical evidence with randomized controlled trials. Ongoing intervention programs are needed to treat hypertension, diabetes and dyslipidemia until the goals are met. Lifestyle changes including smoking cessation, weight reduction and physical activity need to be emphasized regularly.

Based on available evidence, all patients with any of the following risk factors - age ≥50 years, angina, diabetes, prior CVA, MI or PVD should be considered candidates for non invasive cardiac screening regardless of symptoms. Lower threshold for screening may be needed while dealing with patients with low functional capacity (< 4 METS). No adequate data are available at this time for recommendations for CV screening solely based on non traditional risk factors in this group, i.e. hyperhomocys-teinemia, hyperuricemia, high calcium phosphorus product, high C reactive protein. DSE can be an ideal initial screening modality in the absence of contraindications as it also provides assessment of the cardiac chambers, valves and LVH. It is important that adequate stress levels are reached to avoid false negative results. Holding betablockers for 24 hours prior to testing if not contraindicated can improve the ability to achieve target heart rate. Incomplete tests due to severe hypertensive response or submaximal heart rates need to be followed by other tests (e.g. adenosine nuclear or CA). Lower threshold to do CA is needed in patients with low LVEF to diagnose ischemia as the cause of decreased LVEF.

CV risk screening for KT is unique in that it needs to assess not only the perioperative risk, but also should ideally assess the CV risk beyond that period and into the early years of transplantation in order for these patients to benefit from the whole process. As revascularizable disease remains under 10-15% in most series performing CA, despite the high cardiac events and mortality in this group, the answer may very well lie in life style modification, risk reduction and aggressive medical therapy until pre defined goals are met.

## ABBREVIATIONS

ACC/AHA: = American College or Cardiology/ American Heart Association
CV: = Cardiovascular
CKD: = Chronic kidney disease
KT: = Kidney transplantation
CAD: = Coronary artery disease
DSE: = Dobutamine stress echocardiography
SPECT: = Single photon emission computed tomography
CA: = Coronary angiography
EKG: = Electrocardiogram
MI: = Myocardial infarction
DM: = Diabetes mellitus
LDL: = Low density lipoprotein
PVD: = Peripheral vascular disease
